# Identification and Characterization of Dipeptidyl Peptidase-IV Inhibitory Peptides from Oat Proteins

**DOI:** 10.3390/foods11101406

**Published:** 2022-05-12

**Authors:** Wei Wang, Xiaoqing Liu, Yiju Li, Haixi You, Zhipeng Yu, Liying Wang, Xuebo Liu, Long Ding

**Affiliations:** 1 College of Food Science and Engineering, Northwest A&F University, Xianyang 712100, China; Wangwei0402@nwafu.edu.cn (W.W.); xiaoqingliu33@163.com (X.L.); liyiju@nwafu.edu.cn (Y.L.); haixiyou@nwafu.edu.cn (H.Y.); wly99@nwafu.edu.cn (L.W.); xueboliu@nwsuaf.edu.cn (X.L.); 2 College of Food Science and Engineering, Bohai University, Jinzhou 121013, China; yuzhipeng20086@sina.com; 3 Engineering Research Center of Grain and Oil Functionalized Processing, Universities of Shaanxi Province, Xianyang 712100, China

**Keywords:** oats, polypeptide bioactive, dipeptidyl peptidase-IV, molecular docking

## Abstract

In this study, flavourzyme, papain, neutrase, and alcalase, as well as gastrointestinal digestion simulated with pepsin and pancreatin, were used to hydrolyze oat protein, and the dipeptidyl peptidase-IV (DPP-IV) inhibitory activities of the oat protein hydrolysates were investigated. The results indicated that the oat protein hydrolysate by neutrase showed the most potent DPP-IV inhibitory property with an IC_50_ value of 2.55 ± 0.38 mg/mL. Using UPLC-MS/MS, ten new DPP-IV inhibitory peptides were identified from the oat protein hydrolysate by neutrase. Among these peptides, IPQHY, VPQHY, VAVVPF, and VPLGGF exhibited the strongest DPP-IV inhibitory activity with IC_50_ values below 50 μM, and all of them acted as mixed-type inhibitors. Molecular docking indicated that the above four oat-derived peptides were predicted to form hydrogen bonds, attractive charge, and hydrophobic interactions with the residues of the active site of DPP-IV. Therefore, our results suggest that oat is an excellent protein source for food-derived DPP-IV inhibitory peptides and it has the prospect of becoming a dietary supplement for T2DM.

## 1. Introduction

Diabetes mellitus (DM), a metabolic syndrome, is one of the most prominent health problems in the world. Most diabetes can be divided into two main etiological mechanisms. Type I diabetes mellitus (T1DM) is an autoimmune disease in which absolute insulin deficiency due to the immune-mediated destruction of beta cells in the pancreas leads to hyperglycemia. However, insulin resistance is the primary feature of type 2 diabetes mellitus (T2DM) and can be prevented by interventions of lifestyle and diet [1]. After eating, the concentration of endogenous active glucagon-like peptide-1 (GLP-1) in the plasma increases two to three times, which enhances about 60% of the dietary-induced insulin secretion from the beta cells of the islet and plays an important role in glucose homeostasis [2]. Unfortunately, GLP-1 can be quickly inactivated by the enzyme of dipeptidyl peptidase IV (DPP-IV), which primarily cleaves the N-terminal dipeptide from the peptides, leading to incretin deficiency and hyperglycemia [3]. This implies the importance of DPP-IV inhibitors in the management of T2DM by protecting incretin effects and enhancing insulin secretion.

It is known that synthetic DPP-IV inhibitors used in clinics are often reported to have various side effects, such as headache, hypoglycemia, pancreatitis, arthralgia, and infections [4]. As a consequence, the identification of novel efficient and safe DPP-IV inhibitors from food has earned considerable interest [5]. It has been found that the many peptides produced from the enzymatic hydrolysis of dietary proteins exhibit excellent DPP-IV inhibitory activities and show potential as a glucose-lowering therapy, with less adverse effects than synthetic drugs in T2DM [6]. Milk proteins are the most reported source of food-derived DPP-IV inhibitory peptides [7]. However, given the challenges regarding environmental concerns and food safety, the exploration of plant food protein-derived antidiabetic peptides is increasing. Currently, the hydrolysates and peptides derived from wheat gluten, rapeseed, quinoa, and soybean proteins have all been proven to show DPP-IV inhibitory activities [8,9,10,11].

As one of the most widely cultivated cereal grains, oat contains 12% to 20% protein, higher than many other kinds of cereal [12]. Oat protein comprises 50% to 80% globulins, 4% to 15% prolamins, 1% to 12% albumins, and less than 10% glutenins [13]. Oat-protein-derived peptides have been found to have many biological activities, such as antioxidant and antihypertensive activities [14,15]. However, the DPP-IV inhibitory property of oat-derived peptides has been less investigated.

In this study, the oat proteins were hydrolyzed using different enzymes and the DPP-IV inhibitory activities of the oat protein hydrolysates were determined using glycyl-prolyl-7-amino-4-methyl coumarin (Gly-Pro-AMC) as the substrate. The peptide sequences of the oat protein hydrolysate were further analyzed using an ultra-performance liquid chromatography−tandem mass spectrometer (UPLC-MS/MS). Additionally, the type of inhibition of oat-derived DPP-IV inhibitory peptides and their interactions with the active site of DPP-IV were further explored.

## 2. Materials and Methods

### 2.1. Chemicals

The oats were provided by Lvba Grain and Oil Group Co., Ltd. (Zhangjiakou, Hebei, China). Flavourzyme, papain, neutrase, and alcalase were purchased from Pangbo Biological Engineering Co., Ltd. (Nanning, Guangxi, China). The 2,4,6-trinitrobenzene sulfonic acid (TNBS) was purchased from Huaxia Reagent Co., Ltd. (Chengdu, Sichuan, China). Pepsin (from porcine gastric mucosa, 3200 U/mg), pancreatic (from porcine pancreas, 3 × USP specifications), Tris-HCl, human DPP-IV, acetonitrile (ACN), and formic acid (FA) were purchased from Sigma-Aldrich (St. Louis, MO, USA). Acetic acid was purchased from Acro Biosystems Co., Ltd. (Beijing, China). L-Leu and HEPES were purchased from Solarbio Technology Co., Ltd. (Beijing, China). Gly-Pro-AMC was purchased from AAT Bioquest (Sunnyvale, CA, USA). All of the other chemicals were of analytical grade.

### 2.2. Protein Extraction from Oats

The oat powder was mixed with water in a 1:10 (*w*/*v*) ratio, and the pH was adjusted to 10.5 with 1 M NaOH. After stirring for 2 h, the supernatant was collected after centrifugation at 4000× *g* for 20 min at room temperature. Next, 1 M HCl was used to bring the pH of the supernatant down to 4.3, and the mixture was then agitated for 10 min. After centrifugation at 7000× *g* for 20 min at room temperature, the protein precipitate was recovered, vacuum freeze-dried, and stored at −20 °C.

### 2.3. Preparation of Oat-Protein-Derived Peptides

The oat protein was dissolved in distilled water at a concentration of 5% (*w*/*v*), and heated to 90 °C for 10 min to denature the protein. After cooling down, the pH of the oat protein solution was adjusted with 1 M NaOH or HCl. Flavourzyme, papain, neutrase, or alcalase at an enzyme/substrate ratio of 4% (*w*/*w*) was added to hydrolyze the oat protein for 180 min at the proper temperature (neutrase: 45 °C; flavourzyme: 50 °C; alcalase: 50 °C; papain: 55 °C) and pH (neutrase: 7; flavourzyme: 7; alcalase: 8; papain: 7). In addition, in vitro simulated gastrointestinal digestion (SGID) of the oat protein by pepsin (pH 2.0, 37 °C, 90 min) and pancreatin (pH 7.0, 37 °C, 240 min) was also performed. Pepsin and pancreatin were added at a ratio of 2%. Boiling for 5 min was used to inactivate the enzymes. The hydrolysate was centrifuged at 1300 *g* for 10 min. Then, the supernatant was fractionated by an ultrafiltration membrane with a molecular weight (MW) cut-off of 1000 Da (Shanghai Laungy Membrane Separation Equipment Engineering Co., Ltd., Shanghai, China). The fraction with MW less than 1000 Da was finally collected, freeze-dried, and stored at −20 °C.

### 2.4. Determination of Amino Nitrogen Content

The TNBS method was used to measure the amino nitrogen content of the sample, with minor modifications [16]. In brief, a 5 μL sample (0.5 mg/mL) was mixed with 40 μL PBS (0.2125 M, pH 8.2) and 40 μL TNBS (0.1%), and incubated at 50 °C and kept in the dark for 60 min. Then, 80 μL HCl (0.1 M) was added to terminate the process. The absorbance of the mixture at 340 nm was monitored using a microplate reader (BioTek Instruments, Winooski, VT, USA). The standard reagent of L-Leu was used. The amino nitrogen content of the sample was determined as follows:

(1) $$\mathrm{Amino}\;\mathrm{nitrogen}\;\mathrm{content}\;(\mathrm{mmol}\;\mathrm{Leu}/g\;\mathrm{sample})=\frac{{(C}_{s}{\;-\;C}_{b})\times V}{m}$$

where C_s_ and C_b_ are the Leu equivalent concentrations (mM) of the sample and blank groups, respectively. V (L) and m (g) are the volume and mass of the sample used, respectively.

### 2.5. DPP-IV Inhibitory Activity Assay

The DPP-IV inhibitory activity assay of the sample was performed using Gly-Pro-AMC as the substrate, as previously described [17]. Briefly, 50 µL Gly-Pro-AMC (0.2 mM) was added to a 40 µL sample (dissolved in 100 mM HEPES buffer) and incubated for 10 min at 37 °C. The reaction was initiated by adding 10 µL DPP-IV enzyme solution (10 U/L). After being incubated for 60 min at 37 °C, the reaction was terminated by adding 100 µL sodium acetate buffer (1 M, pH 4.0). The fluorescence intensity of the mixture was measured with an excitation wavelength of 350 nm and an emission wavelength of 440 nm. The control group used the HEPES buffer instead of the sample. The blank group used the HEPES buffer instead of the sample and DPP-IV enzyme solution. The DPP-IV inhibitory activity of the sample was calculated as follows:

(2) $$\mathrm{DPP}\text{-}\mathrm{IV}\;\mathrm{inhibitory}\;\mathrm{activity}\;(\%)=\frac{{(A}_{c}-{\;A}_{b}{)\;-\;(A}_{s}-A_{b})}{{(A}_{c}-A_{b})}\times 100\%$$

where A_s_, A_c_, and A_b_ are the intensity of the fluorescence of the sample, control, and blank groups, respectively.

The half-maximal inhibitory concentration (IC_50_) of the sample was calculated according to the DPP-IV inhibition activity against the concentrations of the sample. In addition, the type of inhibition of the sample was obtained from the Lineweaver–Burk equation and double reciprocal plots.

### 2.6. UPLC-MS/MS

The sample dissolved in 0.1% FA was analyzed in an Ultimate 3000 UPLC system (Thermo Scientific, Waltham, MA, USA) using a pre-column of Acclaim PepMap RPLC C_18_ column (300 μm × 5 mm, 100 Å, 5 μm; Thermo Scientific) and an analytical nano-column of Acclaim PepMap RPLC C_18_ column (150 μm × 150 mm, 100 Å, 1.9 μm; Thermo Scientific). Gradient elution was used with a mobile A of 0.1% FA in 2% ACN and a mobile B of 0.1% FA in 80% ACN. Mobile B increased from 6% to 9% in 5 min, 9% to 14% in 15 min, 14% to 30% in 30 min, 30% to 40% in 8 min, and 40% to 95% in 2 min. The flow rate was set at 600 nL/min. The sample was then analyzed using Q Exactive Hybrid Quadrupole-Orbitrap Mass Spectrometry (Thermo Scientific). The spray voltage and the capillary temperature were set at +2.2 kV and 270 °C. The m/z for the full scan was set from 100 to 1200. The scan resolutions for MS and MS/MS were set to 70,000 and 17,500, respectively. The normalized collision energy setting was set at 40. The raw MS data file was analyzed and searched against the database of UniProt *Avena sativa* L. (Oat) using Byonic. The precursor ion mass tolerance and MS/MS tolerance were set at 20 ppm and 0.02 Da, respectively. Only highly confidently recognized peptides were used.

### 2.7. Molecular Docking

The interactions between the oat-derived peptides and human DPP-IV were investigated by the molecular docking technique using Discovery Studio 2017 client software. The X-ray diffraction crystal structure of human DPP-IV was obtained from the RCSB Protein Database (PDB code: 2BGR). After removing water and adding hydrogen atoms, the structure of human DPP-IV was defined as the receptor. The structures of oat-derived DPP-IV inhibitory peptides were defined as the ligands after the energy was minimized using a CHARMm force field. The CDOCKER protocol was used for molecular docking with a binding site of coordinates x = 39.964044, y = 51.623473, and z = 38.513685, and a radius of 9 Å. The best binding conformation was output according to CDOCKER energy. The receptor–ligand interaction analysis was further used to explore the interactions between the peptides and DPP-IV.

### 2.8. Statistical Analysis

All data were presented in the form of mean ± standard deviation (*n* = 3). The difference between two groups was analyzed using Student’s t-test, and the difference between three and more groups was analyzed using one-way analysis of variance (ANOVA) with the least significant difference (LSD) test. The statistical analysis was performed using SPSS 19.0 software. The significance level was *p* < 0.05.

## 3. Results and Discussion

### 3.1. Amino Nitrogen Contents and DPP-IV Inhibitory Activities of Oat Protein Hydrolysates

In this study, the amino nitrogen contents of the oat protein hydrolysates by different enzymes were determined using the TNBS method. A higher amino nitrogen content represented a higher degree of hydrolysis. The results revealed that the oat hydrolysate by neutrase had the highest amino nitrogen content, followed by papain (*p* < 0.05), both of which were higher than the other enzymes and SGID (*p* < 0.05), as shown in Table 1. This suggested that the oat hydrolysates by neutrase and papain had a relatively higher degree of hydrolysis.

**Figure 1:**
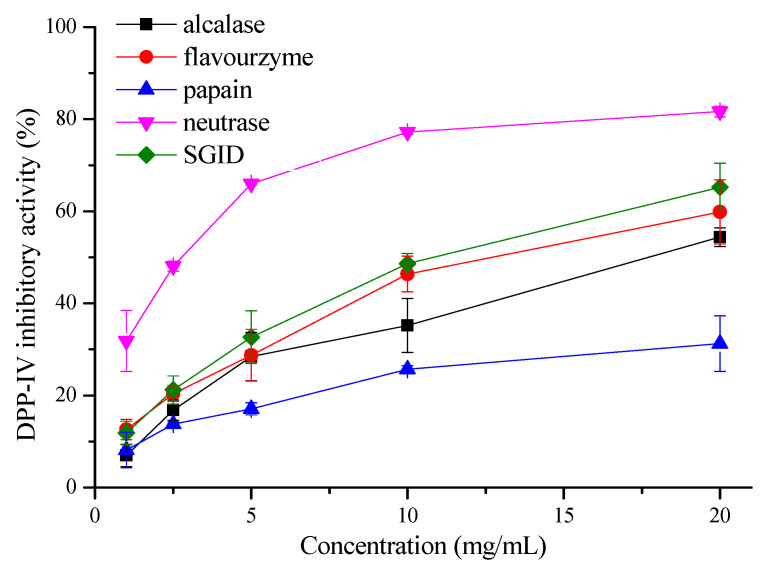
DPP-IV inhibitory activities of the oat protein hydrolysates by different enzymes.

The DPP-IV inhibitory activities of the oat protein hydrolysates by different enzymes with Gly-Pro-AMC as the substrate are shown in Figure 1. It was found that the oat protein hydrolysates by different enzymes all displayed DPP-IV inhibitory activities at final concentrations ranging from 1 to 20 mg/mL, with a significant dose–effect (*p* < 0.05). However, the hydrolysate by neutrase exhibited the highest DPP-IV inhibitory activity with an IC_50_ of 2.55 ± 0.38 mg/mL compared with that of other enzymes (*p* < 0.05). At a concentration of 20 mg/mL, the DPP-IV inhibitory activity of the oat hydrolysate by neutrase was determined to be 81.70% ± 1.18%. In addition, the oat hydrolysates by SGID and flavourzyme also showed high activities, with an IC_50_ of 10.55 ± 1.24 mg/mL and 14.20 ± 5.64 mg/mL, respectively, followed by alcalase, with an IC_50_ of 20.34 ± 5.44 mg/mL (*p* < 0.05) (Table 1). In contrast, the oat hydrolysate by papain displayed the lowest activity.

The DPP-IV inhibitory property of the oat hydrolysate has also been reported elsewhere. For example, the tryptic hydrolysate of oat globulin was found to inhibit DPP-IV activity, with an IC_50_ of 2.04 mg/mL [18], which was comparable to the oat hydrolysate by neutrase in our study. The oat hydrolysates by alcalase and papain have also been found to show a DPP-IV inhibitory activity of about 50% at a concentration of 0.5 mg/mL and 1 mg/mL, respectively [19]. In addition, the oat hydrolysate by SGID was found to have a high DPP-IV inhibitory activity with an IC_50_ of 0.99 mg/mL [20]. What should be noted is that the activities of the oat hydrolysate by SGID, alcalase, and papain above were all higher than that in our present study. This may be because of the difference in oat raw material, hydrolysis conditions, and the DPP-IV inhibitory activity assay method. In this study, oat proteins were hydrolyzed by four enzymes—neutrase, flavourzyme, alcalase, papain, and SGID. The neutrase hydrolysate showed the highest degree of hydrolysis and DPP-IV inhibitory activity.

### 3.2. DPP-IV Inhibitory Activities of Oat-Protein-Derived Peptides

**Figure 2:**
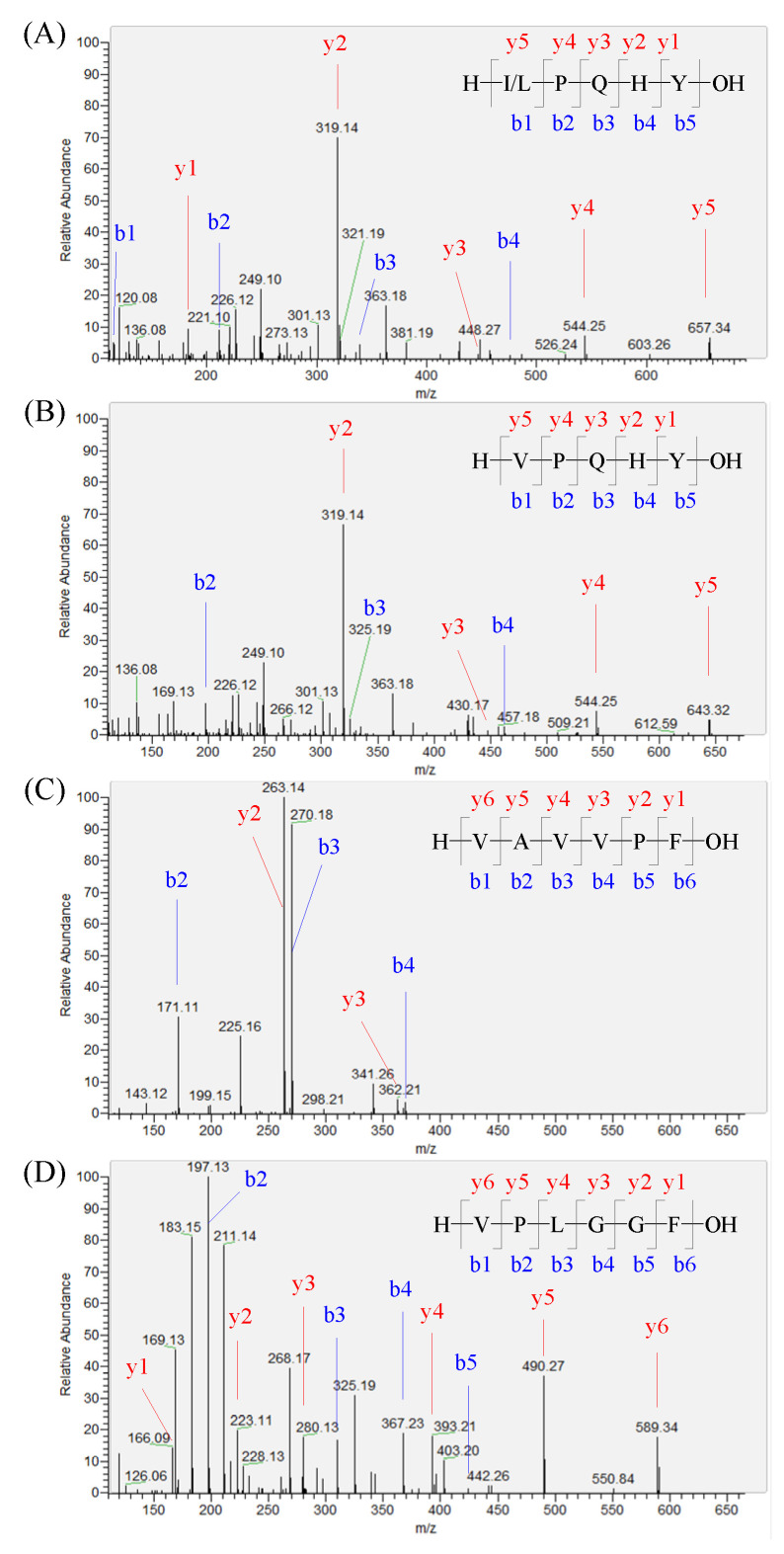
Identification of the oat-protein-derived peptides. The MS/MS spectra of the precursor ions with m/z at 657.33, 643.32, 631.38, and 589.33 were determined to be IPQHY/LPQHY (**A**), VPQHY (**B**), VAVVPF (**C**), and VPLGGF (**D**), respectively.

The peptide sequences of the oat hydrolysate by neutrase were identified using UPLC-MS/MS. In total, 710 peptides with 5 to 10 amino acids were successfully identified. It is well known that the peptide containing a Pro or Ala at the second position of the N-terminus and a hydrophobic amino acid residue at the N-terminus usually shows a DPP-IV inhibitory activity [7]. In addition, the active site of DPP-IV is located in a cavity with two narrow side openings, allowing small substrates or inhibitors to enter [21]. In the last decade, most of the demonstrated food-derived DPP-IV inhibitory peptides had a relatively low MW. In this study, 46 oat peptides had a Pro or Ala at the second position of the N-terminus and a hydrophobic amino acid residue at the N-terminal position, and 18 of them had a low MW with five and six amino acids in length. Finally, 10 peptides, IPQHY, VPQHY, VAVVPF, VPLGGF, LPQHY, VAEVPF, PPHCP, MAGQVF, AAVPF, and LPQYQA, were demonstrated to show DPP-IV inhibitory activities. The identification information of these peptides is summarized in Table 2 and the MS/MS spectra are shown in Figure 2 and Figure S1.

Among the 10 novel peptides, only LPQYQA showed a relatively low activity, whereas the other nine peptides (IPQHY, VPQHY, VAVVPF, VPLGGF, LPQHY, VAEVPF, PPHCP, MAGQVF, and AAVPF) all exhibited superior DPP-IV inhibitory activities with a significant dose−effect at concentrations ranging from 25 μM to 400 μM (*p* < 0.05, Table 3). In particular, IPQHY, VPQHY, VAVVPF, and VPLGGF had the highest DPP-IV inhibitory activities, with an IC_50_ of 25.72 ± 1.06 μM, 30.78 ± 1.93 μM, 33.91 ± 4.83 μM, and 40.22 ± 0.95 μM, respectively; followed by LPQHY, VAEVPF, and PPHCP, with an IC_50_ of 62.66 ± 71.25 μM, 65.33 ± 13.08 μM, and 77.14 ± 2.75 μM, respectively; and MAGQVF of 223.31 ± 6.16 μM and AAVPF of 282.31 ± 27.18 μM, respectively (*p* < 0.05).

As discussed above, the DPP-IV inhibitory properties of the oat hydrolysates by different enzymes of alcalase, flavourzyme, papain, trypsin, and SGID have been demonstrated previously [18,19,20,22]. However, the sequences of DPP-IV inhibitory peptides from the oat proteins have been rarely identified. Only a DPP-IV inhibitory peptide LQAFEPLR from the tryptic hydrolysate of oat globulin was identified, and its DPP-IV inhibitory activity was determined with an IC_50_ of 103.5 μM [20]. What should be noted is that the present work demonstrated the DPP-IV inhibitory activity of the oat protein hydrolysate by neutrase for the first time and identified 10 novel oat-derived DPP-IV inhibitory peptide sequences. The oat-derived peptides IPQHY, VPQHY, VAVVPF, and VPLGGF all showed excellent DPP-IV inhibitory activities with an IC_50_ less than 50 μM, and the peptides LPQHY, VAEVPF, and PPHCP also had high DPP-IV inhibitory activities with an IC_50_ ranging from 60 μM to 80 μM.

IPI and VPL were the most known DPP-IV inhibitory peptides, with IC_50_ values of 3.22 and 16.8 μM, respectively [23]. It is noted that, different from the substrate of glycyl-prolyl-p-nitroanilide hydrochloride (Gly-Pro-pNA), a fluorogenic substrate of Gly-Pro-AMC was used in the present study. Nevertheless, IPI was used as a positive control in our study. The results revealed that, when using Gly-Pro-AMC as the substrate, IPI still exhibited a strong DPP-IV inhibitory activity with an IC_50_ of 5.95 ± 0.91 μM, similar to the reported value of 3.22 μM with Gly-Pro-pNA as the substrate. This indicated the reliability of the determination method of the DPP-IV inhibitory activity assay in our study.

It is known that a number of food-protein-derived peptides show DPP-IV inhibitory activities. For example, whey-protein-derived IPA and IPAVF [24,25], as well as casein-derived IPIQY and LPVPQ, have shown DPP-IV inhibitory activities with an IC_50_ ranging from 30 μM to 50 μM [26,27], comparable to that of the oat-derived peptides IPQHY, VPQHY, VAVVPF, and VPLGGF in the present study. The rapeseed-derived IPQVS, camel milk protein-derived LPVP, and MPVQA showed DPP-IV inhibitory activities with an IC_50_ ranging from 50 μM to 100 μM [9,28], which were similar to the oat-derived peptides LPQHY, VAEVPF, and PPHCP in our study. It was noted that more of the food-derived peptides only showed relatively low DPP-IV inhibitory properties with an IC_50_ over 100 μM and even 200 μM. In a previous study, the wheat gluten hydrolysate by flavourzyme was determined to show a high DPP-IV inhibitory activity with an IC_50_ of 1.25 ± 0.09 mg/mL. However, the identified wheat-derived peptides, such as MPF and VAVPV, only showed a relatively low DPP-IV inhibition with an IC_50_ of about 500 μM [8], which indicated that oat protein was a good source for preparing DPP-IV inhibitory peptides.

### 3.3. Inhibition Modes of Oat-Derived DPP-IV Inhibitory Peptides

**Figure 3:**
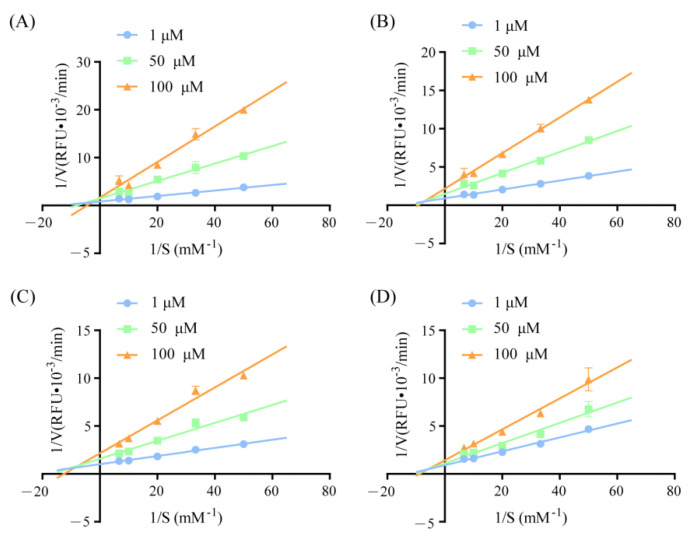
Inhibition mode of peptide IPQHY (**A**), VPQHY (**B**), VAVVPF (**C**), and VPLGGF (**D**) on DPP-IV.

An enzyme-kinetics experiment was used to further investigate the inhibition modes of the oat-derived DPP-IV inhibitory peptides IPQHY, VPLGGF, VPQHY, and VAVVPF. According to the results from plotting the Lineweaver−Burk double-reciprocal, oat-derived peptides IPQHY, VPLGGF, VPQHY, and VAVVPF all behaved as competitive−noncompetitive mixed-type inhibitors on DPP-IV (Figure 3). These results indicate that the four oat-derived peptides exhibited a DPP-IV inhibitory activity by binding to not only the active site, but also outside the catalytic center of DPP-IV. Similar results have also been reported for the *Chlorella* *Vulgaris* protein-derived IPR and VPW [29]; camel milk protein-derived SPVVPF and ILDKVGINY [28]; and *Ruditapes* *Philippinarum* protein-derived FAGDDAPR, FAGDDAPRA, and FLMESH [30].

### 3.4. Molecular Interactions between Oat-Derived Peptides and the Active Site of Human DPP-IV

**Figure 4:**
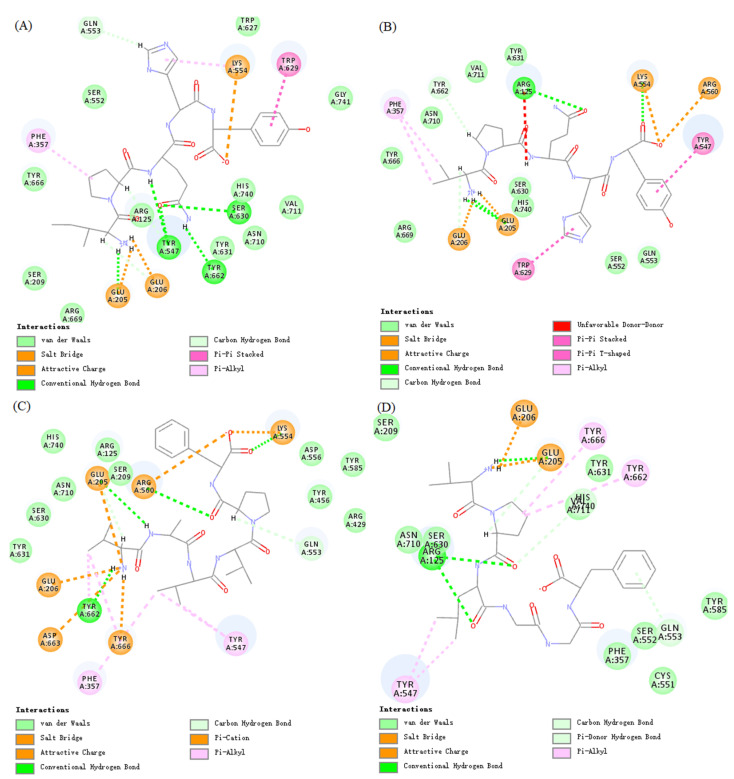
Molecular interactions between DPP-IV and peptides IPQHY (**A**), VPQHY (**B**), VAVVPF (**C**), and VPLGGF (**D**).

To explore the interactions of the oat-derived DPP-IV inhibitory peptides of IPQHY, VPLGGF, VPQHY, and VAVVPF with the active site of DPP-IV, molecular docking was conducted. The –CDOCKER energy of IPQHY, VPLGGF, VPQHY, and VAVVPF was determined to be 94.32 kcal/mol, 93.76 kcal/mol, 93.83 kcal/mol, and 90.74 kcal/mol, respectively, and the –CDOCKER interaction energy was 93.45 kcal/mol, 85.56 kcal/mol, 84.84 kcal/mol, and 74.83 kcal/mol, respectively. It was observed that the –CDOCKER energy, especially the –CDOCKER interaction energy of IPQHY, VPLGGF, VPQHY, and VAVVPF, was consistent with the in vitro DPP-IV inhibitory activities. The interactions between peptides of IPQHY, VPLGGF, VPQHY, and VAVVPF and human DPP-IV are shown in Figure 4. IPQHY, VPLGGF, VPQHY, and VAVVPF were predicted to form multiple interactions with the prominent residues of the active site of DPP-IV. For example, IPQHY formed three attractive charge interactions with Glu205, Glu206, and Lys554; ten hydrogen bond contacts with Glu205, Glu206, Tyr547, Gln553, Ser630, and Tyr662; and four hydrophobic interactions with Phe357, Lys554, and Trp629 of DPP-IV. Similar results were also observed for VPQHY, VAVVPF, and VPLGGF.

IPI was also docked to DPP-IV as a positive control in this study. It was found that the –CDOCKER energy and –CDOCKER interaction energy of IPI were 58.62 and 65.45, respectively, which are relatively lower than those of the oat-derived peptides IPQHY, VPLGGF, VPQHY, and VAVVPF. However, IPI formed more interactions with DPP-IV, including five attractive charge interactions with the residues of Arg125, Glu205, and Glu206; ten hydrogen bond interactions with the residues of Arg125, Glu205, Glu206, and Tyr662; and five hydrophobic interactions with the residues of Phe357, Tyr547, Tyr662, and Tyr666. What should be noted is that, on the one hand, a peptide with a longer length had more active groups and was speculated to form more interactions with the receptor. On the other hand, it was essential to analyze the critical role of the residues forming interactions with the peptide for the activity of the receptor.

It is known that the active site of human DPP-IV contains an S1 pocket (Tyr631, Val656, Trp659, Tyr662, Tyr666, and Val711) and an S2 pocket (Arg125, Glu205, Glu206, and Phe357) [31]. The S1 pocket is hydrophobic and shaped for the side chain of Pro or Ala of peptides, which determines the specificity of DPP-IV. In addition, the NH_3_^+^ group at the N-terminus of the peptide is predominantly recognized by the key residues of the S2 pocket, such as Glu205 and Glu206, mainly by hydrogen bonds and attractive charge interactions [32]. The current work suggests that the oat-derived peptides IPQHY, VPQHY, VPLGGF, and VAVVPF formed hydrophobic and hydrogen bond interactions with the residues of Tyr662 and Tyr666 in the S1 pocket. Aside from various charge interactions, these four peptides also formed hydrogen bond interactions with the residues of Arg125, Glu205, and Glu206 and hydrophobic interactions with the residue of Pher357 in the S2 pocket. All of these may contribute to the superior DPP-IV inhibitory effects of the oat-protein-derived IPQHY, VPQHY, VPLGGF, and VAVVPF.

## 4. Conclusions

The oat protein hydrolysate by neutrase had the highest degree of hydrolysis and the highest DPP-IV inhibitory capacity. From the oat protein hydrolysate by neutrase, 10 peptides were identified with DPP-IV inhibitory activities. Among them, IPQHY, VPQHY, VAVVPF, and VPLGGF showed the strongest inhibition activities and all behaved as mixed-type inhibitors. In addition, IPQHY, VPQHY, VAVVPF, and VPLGGF were predicted to form multiple interactions, including hydrogen bonds, attractive charge, and hydrophobic interactions with the active site of DPP-IV. This work demonstrated that oat protein is a good source for food-derived DPP-IV inhibitory peptides and has the potential for the management of T2DM.

## Figures and Tables

**Table 1:** Amino nitrogen contents and DPP-IV inhibitory activities of oat protein hydrolysates by different enzymes. The data marked by different letters for each determination are significantly different (*p* < 0.05).

Oat Hydrolysates by Different Enzymes	Amino Nitrogen Content (mmol Leu/g Sample)	DPP-IV Inhibitory Activity (IC_50_, mg/mL)
alcalase	7.77 ± 0.68 ^a^	20.34 ± 5.44 ^c^
flavourzyme	8.28 ± 0.84 ^a^	14.20 ± 5.64 ^b^
papain	29.14 ± 4.72 ^b^	nc
neutrase	35.79 ± 7.19 ^c^	2.55 ± 0.38 ^a^
SGID	11.27 ± 2.01 ^a^	10.55 ± 1.24 ^b^

nc indicates that the IC_50_ was not calculated.

**Table 2:** Identification information of the oat-protein-derived peptides.

Sequence	Mass (Da)	m/z	Retention Time (min)	Protein Source (Protein Name)	Accession No.	Position
IPQHY	656.33	657.33	37.75	12S globulin	P12615	421–425
LPQHY	656.33	657.33	37.75	12S globulin	O49257	374–378
VPQHY	642.31	643.32	33.44	12S globulin	O49258	417–421
VAEVPF	660.35	661.36	45.58	Avenin	I4EP88	71–76
LPQYQA	718.37	719.37	28.42	Avenin	I4EP76	147–152
				Avenin protein	F4MJY6	166–171
MAGQVF	651.31	652.31	36.97	Avenin	I4EP76	136–141
				Avenin protein	F4MJY5	151–156
				Gliadin-like avenin	L0L837	137–142
PPHCP ^a^	606.26	607.26	24.6	Avenin protein	G8ZCW3	228–232
				Gliadin-like avenin	L0L6J0	268–272
AAVPF	503.27	504.28	39.02	Avenin protein	G8ZCW3	114–118
				Gliadin-like avenin	L0L6J0	112–116
VAVVPF	630.37	631.38	50.28	Gliadin-like avenin	L0L5G8	84–89
				Avenin protein	G8ZCW0	90–95
VPLGGF	588.33	589.33	45.58	Gliadin-like avenin	L0L5I0	224–229

^a^ The Cys in peptide PPHCP was modified with carbamidomethylation.

**Table 3:** DPP-IV inhibitory activities of the oat-derived peptides. The data marked by different letters for each determination were significantly different (*p* < 0.05).

Sequence	DPP-IV Inhibitory Activity (%)					DPP-IV Inhibitory Activity (IC_50_, μM)
	25 μM	50 μM	100 μM	200 μM	400 μM	
IPQHY	49.44 ± 1.19 ^a^	66.89 ± 1.46 ^a,b^	82.67 ± 0.74 ^a^	91.02 ± 1.20 ^a^	95.84 ± 0.52 ^a^	25.72 ± 1.06 ^d^
VPQHY	40.55 ± 4.59 ^a,b^	69.27 ± 4.66 ^a^	78.83 ± 2.17 ^a^	87.25 ± 0.25 ^a^	94.30 ± 0.28 ^a,b^	30.78 ± 1.93 ^d^
VAVVPF	42.93 ± 2.31 ^a,b^	61.53 ± 5.19 ^b,c^	65.97 ± 4.07 ^c^	78.86 ± 1.23 ^b^	87.99 ± 0.39 ^d,e^	33.91 ± 4.83 ^d^
VPLGGF	33.99 ± 0.76 ^b^	55.92 ± 1.86 ^c^	74.38 ± 0.88 ^b^	86.71 ± 1.10 ^a^	92.67 ± 0.36 ^b,c^	40.22 ± 0.95 ^d^
LPQHY	21.27 ± 4.86 ^c^	37.36 ± 4.24 ^d^	62.63 ± 1.21 ^c,d^	80.37 ± 4.84 ^b^	89.87 ± 0.41 ^d^	62.66 ± 71.25 ^c^
VAEVPF	33.03 ± 12.30 ^b^	43.43 ± 2.27 ^d^	58.16 ± 3.86 ^d^	74.20 ± 2.32 ^c^	86.18 ± 0.96 ^e^	65.33 ± 13.08 ^c^
PPHCP	4.42 ± 3.95 ^d^	29.92 ± 1.84 ^e^	58.88 ± 1.10 ^d^	81.35 ± 0.48 ^b^	89.29 ± 0.45 ^d^	77.14 ± 2.75 ^c^
MAGQVF	10.40 ± 4.35 ^d^	13.04 ± 2.11 ^f^	26.71 ± 3.49 ^e^	49.40 ± 1.90 ^d^	67.48 ± 2.97 ^f^	223.31 ± 6.16 ^b^
AAVPF	5.42 ± 3.61 ^d^	16.67 ± 3.38 ^f^	28.92 ± 2.62 ^e^	39.92 ± 2.79 ^e^	59.80 ± 2.84 ^g^	282.31 ± 27.18 ^a^
LPQYQA	nd	nd	nd	5.59 ± 3.10 ^f^	29.76 ± 2.50 ^h^	nc

nd indicates that the DPP-IV inhibitory activity is not determined; nc indicates that the IC_50_ value is not calculated.

## Data Availability

The data that support the findings of this study are available from the corresponding author upon reasonable request.

## Supplementary Materials

The following supporting information can be downloaded at https://www.mdpi.com/article/10.3390/foods11101406/s1, Figure S1. MS/MS spectra of oat-derived peptides VAEVPF, PPHCP, MAGQVF, AAVPF, and LPQYQA.
