# Supplementary material for: Identification and Characterization of Dipeptidyl Peptidase-IV Inhibitory Peptides from Oat Proteins
Source: Foods. 2022 May 12;11(10):1406. doi: 10.3390/foods11101406 (PMC9141920; doi:10.3390/foods11101406)
Supplement: Supplementary file 1 [file foods-11-01406-s001.zip › foods-1701078-supplementary-done.pdf]

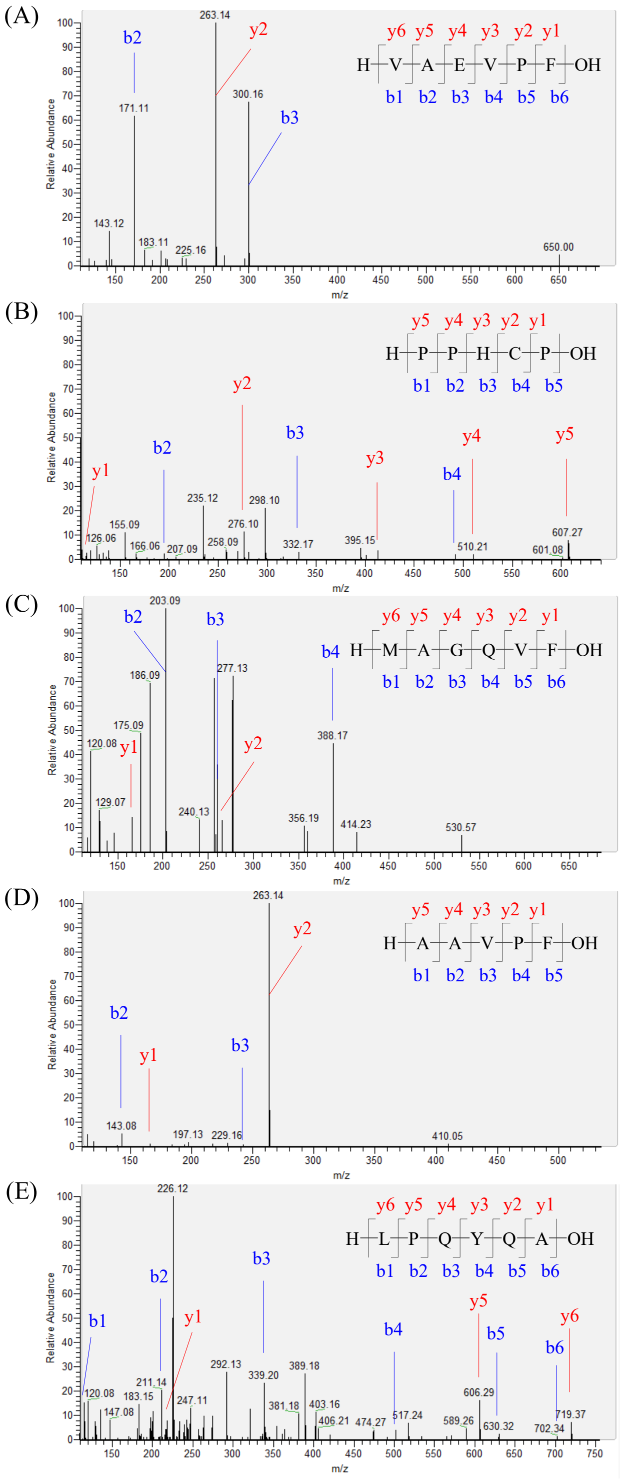

Figure S1. MS/MS spectra of oat-derived peptides VAEVPF, PPHCP, MAGQVF, AAVPF, and LPQYQA. The precursor ions with m/z at 661.36.33, 607.26, 652.31, 504.28, and 719.37 were determined to be peptide VAEVPF (A), PPHC(+57.02)P (B), MAGQVF (C), AAVPF (D), and LPQYQA (E), respectively..
